# Thermodynamics‐Guided High‐Throughput Discovery of Eutectic High‐Entropy Alloys for Rapid Solidification

**DOI:** 10.1002/advs.202401559

**Published:** 2024-06-18

**Authors:** Liuliu Han, Zhongji Sun, Wenzhen Xia, Shao‐Pu Tsai, Xukai Zhang, Jing Rao, Pei Wang, Andrew Chun Yong Ngo, Zhiming Li, Yong Liu, Dierk Raabe

**Affiliations:** ^1^ Max‐Planck‐Institut für Eisenforschung Max‐Planck‐Straße 1 40237 Düsseldorf Germany; ^2^ Institute of Materials Research and Engineering Agency for Science Technology and Research Singapore 138634 Singapore; ^3^ School of Metallurgical Engineering Anhui University of Technology Maanshan 243002 China; ^4^ Department of Materials Science and Engineering National Taiwan University Taipei 10617 Taiwan; ^5^ School of Materials Science and Engineering Central South University Changsha 410083 China; ^6^ State Key Laboratory of Powder Metallurgy Central South University Changsha 410083 China

**Keywords:** additive manufacturing, alloy design, eutectic high‐entropy alloy, rapid solidification

## Abstract

Excellent castability, significantly refined microstructure, and good mechanical properties make eutectic high‐entropy alloys (EHEAs) a natural fit for rapid solidification processes, e.g., additive manufacturing. Previous investigations have focused on developing EHEAs through trial and error and mixing known binary eutectic materials. However, eutectic compositions obtained from near‐equilibrium conditions do not guarantee a fully eutectic microstructure under rapid solidifications. In this work, a thermodynamically guided high‐throughput framework is proposed to design EHEAs for rapid solidification. Empirical formulas derived from past experimental observations and thermodynamic computations are applied and considered phase growth kinetics under rapid solidification (skewed phase diagram). The designed alloy candidate, Co_25.6_Fe_17.9_Ni_22.4_Cr_19.1_Ta_8.9_Al_6.1_ (wt.%), contains nanostructured eutectic lamellar and shows a high Vickers hardness of 675 Hv. In addition to this specific composition, the alloy design toolbox enables the development of new EHEAs for rapid solidification without the limitation of previous knowledge.

## Graphical abstract

The current work proposes a simple approach of using empirical formulas and existing thermodynamic data to discover eutectic high‐entropy alloys for rapid solidification processes, such as metal additive manufacturing. The exemplarily devised material containing face‐center‐cubic and Laves phases demonstrates high hardness in its as‐built state.

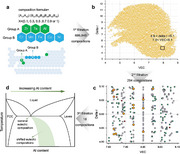

## 1 Introduction

Metal additive manufacturing (AM) provides one‐step material synthesis, heat treatment, and shaping of ready‐to‐use parts for direct industrial deployment.^[^ ^1^ ^]^ Components with ultrahigh hardness in their as‐built state are critical, for instance, in precision instrument, molding, and drilling applications. These parts require high resistance against indentation and abrasion damage, but their often‐complicated shapes make coating and machining challenging.^[^ ^2^ ^]^ Yet, most hard materials cannot be fabricated by AM without experiencing significant cracking, e.g., high‐strength steels,^[^ ^3^, ^4^ ^]^ nickel‐based superalloys,^[^ ^5^ ^]^ and high‐entropy alloys.^[^ ^6^, ^7^ ^]^ This is because elements responsible for mechanical strengthening typically undergo significant partitioning or form high‐volume‐fraction brittle precipitates, increasing the crack‐susceptible temperature range^[^ ^8^ ^]^ or residual stress contents^[^ ^9^ ^]^ during rapid solidification. Process optimization alone cannot resolve such cracking issues, as the problem arises from the intrinsic properties of the materials. Therefore, developing ultrahard, easy‐to‐process materials suitable for rapid solidification is an imminent task.

One possible approach to meeting this goal is to combine the excellent castability of eutectic alloys with the high strength of high‐entropy alloys,^[^ ^10^, ^11^ ^]^ viz., eutectic high‐entropy alloys (EHEAs).^[^ ^12^, ^13^, ^14^ ^]^ Such materials are resistant to hot and cold cracks, which are usually observed during AM fabrication.^[^ ^10^, ^11^ ^]^ For hot cracking, cracks occurring with the presence of a liquid film, the narrow solidification range of eutectic systems reduces crack initiation and growth inside the meshy zone,^[^ ^15^ ^]^ and the high fluidity of eutectics also helps to close any existing opening by liquid back feeding.^[^ ^16^ ^]^ For cold cracking, cracks occurring in a solid state due to tensile residual stresses, the refined dual‐phase eutectic lamellar nanostructures^[^ ^17^ ^]^ can accommodate tensile stresses by strain partitioning between the softer and harder phases.^[^ ^18^ ^]^ Combining these advantages with the ability of AM to manipulate site‐specific thermal histories under a broad range of processing conditions,^[^ ^19^ ^]^ there is substantial unexplored space for microstructure design and property tuning.^[^ ^20^, ^21^ ^]^

Despite the immense potential,^[^ ^22^ ^]^ previous investigations on EHEAs development are mainly based on the approaches of trial‐and‐error,^[^ ^23^, ^24^, ^25^, ^26^ ^]^ mixing of known binary eutectic compositions,^[^ ^27^ ^]^ and the subsequent extension to non‐stoichiometric variants.^[^ ^28^, ^29^, ^30^ ^]^ The requirement of large experimental resources and knowledge of existing binary eutectic compositions inevitably restricts the effectiveness and variety of new EHEAs designs. In addition, eutectic compositions obtained from equilibrium or slow cooling conditions do not guarantee a fully eutectic microstructure under rapid solidifications, i.e., the presence of dendrites when eutectic Al‐Si and Fe‐C alloys are processed under the high cooling rate of chill casting.^[^ ^31^ ^]^

Here, we proposed a new strategy to design hard EHEAs suitable for manufacturing under rapid solidification without prior eutectic compositions. The designed materials target a topologically close‐packed Laves phase for high hardness and a face‐centered‐cubic (FCC) phase for enhanced ductility. In total, the approach embodies three main stages. First, the composition space formulation is based on the empirical assisted (A_x1_A_x2_)(B_x3_B_x4_B_x5_B_x6_) formula, where the selection of elements for groups A and B should facilitate the EHEAs formation. Second, the down‐selection of alloys is based on the empirical constraints of average atomic mismatch (δ*r*), and valence electron concentration (*VEC*) which is favorable for the Laves phase development. After solidification, the selected alloys are interpreted through phase diagrams (Calphad) calculation to understand their phase constituents. Third, only compositions with the desired contents of Laves and FCC phases are further modified to make them compatible with rapid‐solidification production.

## 2 Results

### 2.1 Thermodynamics‐Guided High‐Throughput Sequential Filtration Alloy Design Strategy

**Figure 1:**
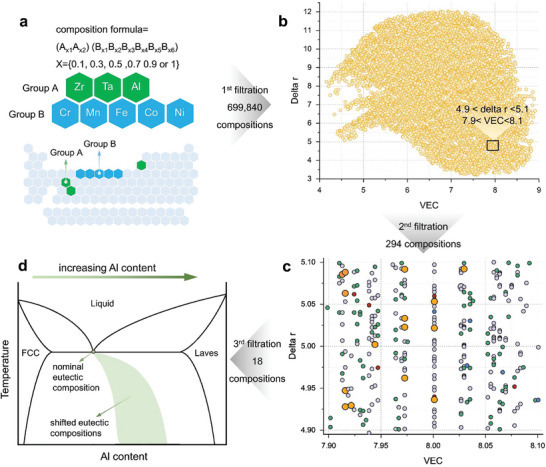
Sequential filtration alloy design strategy for eutectic high‐entropy alloys containing FCC and Laves phases. a) Composition modification based on the chemical formula (A_x1_A_x2_)(B_x3_B_x4_B_x5_B_x6_) and mixing enthalpies. b) 699840 alloys are first selected based on their intrinsic δr and average VEC values (first filtration). c) 294 chemical formulations are further screened to conform to the empirical constraints of 4.9 < δr < 5.1 and 7.9 < VEC < 8.1 (second filtration). Finally, 18 alloys (orange dots) fulfill the phase constitutions (third filtration) and are calculated by thermodynamic simulations (see Methods). The alloys containing 1, 2, 3, and 4 phases are the blue, grey, green, and red dots. d) “Skewed” phase diagram involving “faceted” FCC and “non‐faceted” Laves phases showing hyper‐eutectic compositions are required to enable fully eutectic microstructure under rapid solidification.

The current alloy design strategy is illustrated in **Figure** **1**. The designed (A_x1_A_x2_)(B_x3_B_x4_B_x5_B_x6_) formula is composed of 2 groups of elements, with each taking an independent stoichiometric coefficient of 0.1, 0.3, 0.5, 0.7, 0.9, or 1.0 (Figure 1a). To promote the formation of eutectic microstructures,^[^ ^27^ ^]^ group A elements should have highly negative mixing enthalpies with group B elements, and group B elements have similar mixing enthalpies. In this work, we select Al, Zr, and Ta as group A elements and Cr, Mn, Fe, Co, and Ni as group B elements. Figure 1b shows the formulated 699840 compositions based on their δ*r* and *VEC* values (see Methods).

We next choose compositions within the empirical constraints of 4.9 < δ*r* < 5.1^[^ ^32^ ^]^ and 7.9 < *VEC* < 8.1^[^ ^33^ ^]^ (black frame in Figure 1b), which are reported to favor the formation of the Laves phase. This filtration picks 294 alloys out of the initial 699840 candidates. The Calphad simulation interprets the phase constituents of these 294 alloys. Among them, five are single‐phase, 169 are dual‐phase, 113 have three phases, and seven have four phases. They are represented by the blue, grey, green, and red circles in Figure 1c, respectively. 18 alloys fit our requirements (orange dots in Figure 1c and listed in Table S1) of containing two phases (Laves and FCC) with the potential to form eutectic morphology, and the molar fraction of Laves phase should be higher than 0.2 to guarantee a high hardness.

The formation of eutectic microstructure relies on the cooperative growth of two phases from the liquid, and it is not necessarily equivalent to the equilibrium eutectic (invariant) point.^[^ ^34^ ^]^ For systems consisting of a faceted and a non‐faceted phase, i.e., the Laves and FCC phases, the faceted Laves phase is expected to have slower growth kinetics due to its anisotropic crystal structure and specific growing mechanism.^[^ ^35^, ^36^ ^]^ This difference in their growth kinetics will lead to weak diffusive coupling and render eutectic microstructures difficult to form under rapid solidifications.^[^ ^37^ ^]^ Therefore, additional Laves phase formation elements are needed to compensate for the diffusive coupling, as shown in the “skewed” phase diagram in Figure 1d.

Among the 18 alloys obtained in the final filtration step, we selected the Cr_0.7_Fe_0.7_Co_1.0_Ni_0.9_Al_0.1_Ta_0.3_ candidate (Co_27.0_Fe_18.9_Ni_24.3_Cr_18.9_Ta_8.1_Al_2.7_, wt.%) as representative of the validation of the current alloy design methods and produced pre‐alloyed powder accordingly. This composition is located in the hypoeutectic region through the equilibrium phase diagram calculations. To compensate for the slow growth kinetics of the Laves phase under rapid solidification conditions (“skewed” phase diagram), hypereutectic compositions deviating from the Laves phase regime are desired for a fully eutectic microstructure. Therefore, two more composition constituents (eutectic and hypereutectic) were produced by adding pure Al into the pre‐alloyed powder for further synthesis to confirm our hypothesis. These three alloys are referred to as Hypo‐HEA, E‐HEA, and Hyper‐HEA (Table S2). The microstructure of the powder and the powder blending ratio can be found in Figures S1 and S2.

### 2.2 Microstructures of the as‐Built Alloys

**Figure 2:**
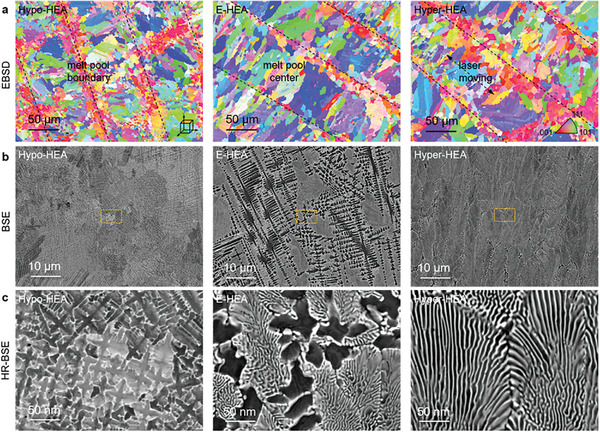
Microstructures of the as‐built alloys with different Al contents from the top surface. a) IPF maps. b) BSE images. c) High‐resolution BSE images showing the enlarged views from the respective framed regions in (b). The left, middle, and right columns correspond to the Hypo‐HEA, E‐HEA, and Hyper‐HEA.

We fabricated these materials by laser powder bed fusion (LPBF). The typical microstructure of the as‐built alloys examined from the top view is shown in **Figure** **2**. No preferred crystallographic texture was observed in the inverse pole figure (IPF) maps (Figure 2a). With an increment in the nominal Al content, the grain size increases continuously from 10 ± 8 µm (Hypo‐HEA) to 15 ± 11 µm (E‐HEA) and then to 18 ± 13 µm (Hyper‐HEA). Figure 2b shows the microstructure at the melt pool centers revealed by backscattered electron (BSE) imaging analysis. Figure 2c shows the enlarged views of those frames, as indicated in Figure 2b.

As expected, the Hypo‐HEA shows a dendritic morphology. The dendritic and interdendritic regions are the FCC and Laves phases, respectively, based on the contrast differences under BSE conditions. The high‐resolution EBSD results (Figure S3) confirm this observation. A mixture of dendritic and eutectic lamellar structures is observed in the E‐HEA (Figure 2 middle column), where the eutectic equilibrium did not yield a fully eutectic microstructure. After further increasing the nominal Al content to Hyper‐HEA, a microstructure mainly composed of fine eutectic lamellar is obtained (Figure 2 right column), agreeing with our prior hypothesis. It should be noted that the Hyper‐HEA is not entirely composed of the eutectic lamella since a small fraction of the dendritic structure is still present (Figure S4). A similar trend in microstructural evolution is observed on the side views of the as‐built samples along their build direction (Figure S5). The crack number density decreases from 13.8 ± 3.2 mm^−2^ (Hypo‐HEA) to 4.7 ± 1.3 mm^−2^ (E‐HEA) and finally to 0.9 ± 0.2 mm^−2^ (Hyper‐HEA) when examined over a total area of 100 mm^2^ for each sample, indicating that the eutectic lamellar is beneficial for crack prevention (Figure S6). No obvious elemental segregation was detected within the as‐built specimens by energy‐dispersive X‐ray spectroscopy (EDS) mappings (Figure S7).

### 2.3 Mechanical Performance of the as‐built Hyper‐HEA with the Lamellar Eutectic Nanostructure

**Figure 3:**
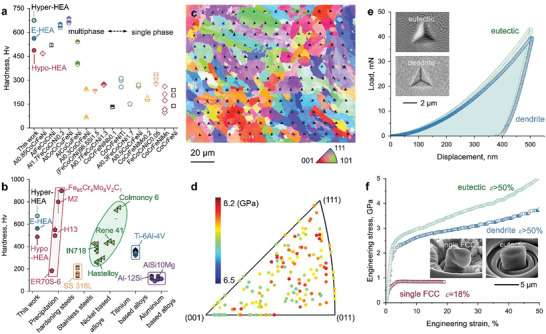
Mechanical performance of the as‐built Hyper‐HEA with the lamellar eutectic nanostructure. a) Hardness of the currently investigated HEAs compared with the established CoCrFeNi‐based HEAs, and b) commercial materials^[^ ^42^, ^43^, ^44^, ^45^, ^46^, ^47^, ^48^, ^49^, ^50^, ^51^ ^]^ by AM. The hollow and semi‐hollow symbols are the measured hardness. The solid symbols are the estimated hardness converted from the yield strength by the empirical relation.^[^ ^52^ ^]^ c) EBSD‐IPF map showing the grain‐orientation dependence of the nanoindentation results for the Hyper‐HEA. d) Corresponding standard stereographic projection showing the hardness values (GPa) from the nanoindentation measurements in crystallographic coordinates. e) Typical load‐displacement curves of the indent from the eutectic and dendrite regions are displayed in the inserted image. f) Micropillar compressive engineering stress–strain curves. The inserted SEM images show the pillars taken from the eutectic and dendrite regions at a total deformation of *ε *= 50% (top left) and the single‐phase CoCrFeNi reference material^[^ ^6^ ^]^ at *ε *= 18% (bottom right), respectively.

The mechanical performance of the current alloys was measured by multiple probing methods, including hardness testing (micro‐ and nanoindentation) and compressive micropillar deformation testing. The Vickers hardness values of the current as‐built materials are compared to other HEAs (**Figure** **3**a) and structural materials (Figure 3b) fabricated by AM. With increasing Al content, the hardness of the as‐built alloys increases monotonously from 487 ± 18 Hv (Hypo‐HEA) to 562 ± 20 Hv (E‐HEA) and reaches a peak value of 675 ± 47 Hv (Hyper‐HEA). Due to the presence of the hard Laves phase, these materials are much harder than most CoCrFeNi‐based HEAs with a single FCC crystal structure, which typically exhibit a hardness value ranging from 150 to 350 Hv.^[^ ^38^ ^]^ Nanoindentation testing was conducted on Hyper‐HEA to elucidate the effect of crystallographic orientation on the hardness property, Figure 3c. 400 indents were tested and the corresponding results are plotted in a stereographic IPF map (Figure 3d). Although the hardness values near the (001) surface plane are generally lower as compared to those close to the (011) and (111) grains, the overall hardness values only vary within a narrow range from 7.7 to 8.1 GPa, indicating a weak effect of crystallographic orientation on hardness. The hardness value of the Hyper‐HEA measured by Vickers hardness measurement (675 Hv) can be converted to 6.62 GPa through Taylor formular^[^ ^39^ ^]^ to assist interpretation and comparison with results recorded by nanoindentation. The size effect likely triggers the difference in hardness due to different testing methods.^[^ ^40^ ^]^ The average hardness values at grain boundaries (7.8 ± 0.3 GPa) are comparable to those within the grains (7.9 ± 0.2 GPa), suggesting that the high hardness of the Hyper‐HEA material is due to the strengthening effects acting inside the individual grains.^[^ ^41^ ^]^

For practical engineering applications, e.g., precision drilling and wear‐resistant coating, the resistance against mechanical indentation and material distortion is important. A representative load‐displacement curve taken close to the (011) crystallographic orientation of the Hyper‐HEA is shown in Figure 3e. The maximum loading force recorded for the eutectic region is ≈42.9 mN, with a penetration depth of 500 nm. No micro‐cracks are detected near the indents (see insets), confirming excellent local damage tolerance. Micropillar compression tests were further conducted to elucidate the deformation behavior of the Laves/FCC eutectic microstructures, Figure 3f. The result is shown together with the LPBF‐built single‐phase (FCC) CoCrFeNi HEA reference material for comparison.^[^ ^6^ ^]^ The yield strength (σ_y_) of the Hyper‐HEA in the eutectic region (2.34 ± 0.21 GPa) is nearly four times that of the CoCrFeNi alloy (0.60 ± 0.05 GPa). Furthermore, a distinct difference in the post‐deformation morphologies is observed between the two materials. The CoCrFeNi sample fails at an engineering strain (ε) of 18% due to the activation of shear bands, which travel across the entire pillar at almost zero work hardening. In contrast, the Hyper‐HEA deforms homogeneously up to ε = 50% under continuous work hardening until the test is stopped manually.

## 3 Discussion

**Figure 4:**
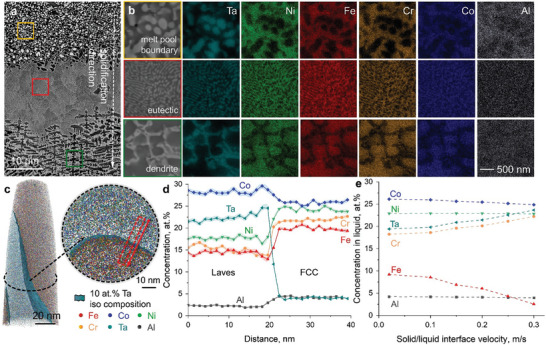
Location‐dependent elemental distribution within melt pools for the Hyper‐HEA. a) BSE overview micrograph showing the microstructure variation near the melt pool boundary. b) Corresponding EDS mappings are taken from the melt pool boundary (orange frame), eutectic (red frame), and dendritic (green frame) regions in (a). c) Atom probe tomography showing the eutectic microstructure. d) Corresponding 1D concentration profile spanning from the Laves phase into the FCC region. e) Effect of solute trapping at different S/L interface velocities on elemental concentrations in the liquid in front of the solidifying S/L interface.

Despite the successful design and synthesis of eutectic microstructures under rapid solidification from the initially devised 699840 compositions, the Hyper‐HEA still has a small fraction (14.5%, Table S3) of non‐eutectic microstructures, mostly present near the melt pool boundaries (Figure S5). Understanding the formation mechanism of these non‐eutectic microstructures is key to the potential production of fully eutectic microstructures under rapid solidification. A representative microstructural evolution across the melt pool boundary of the Hyper‐HEA (**Figure** **4**a) shows that the solidification follows a sequence of i) spherical precipitates, ii) eutectic lamella, and iii) dendrites, from the melt pool boundary to the center.

As eutectic solidification is a diffusion‐controlled process,^[^ ^53^ ^]^ sidewise diffusion (diffusion coupling) synergy between the two coexisting phases during solidification determines the final microstructural feature. The corresponding elemental analysis at the microscale shows that the Laves phase is enriched in Ta (bright contrast), and the remaining dark matrix is FCC (Figure 4b). The overall Ta content shows the most significant variation, with a relative change of 10.2% in atomic concentrations (Figure S8). This value is much higher than the other elements, i.e., Co (1.0%), Ni (2.1%), Cr (0.7%), Fe (1.6%), and Al (2.9%). The Ta concentration is the highest at the melt pool boundary (8.2 wt.%), and it quickly drops to 7.6 and 7.4 wt.% when approaching the melt pool interior. When probing the chemical information of those eutectic lamellar at the near‐atomic scale (Figure 4c), the Laves phase is found to be enriched with Co and Ta (Co_28.8_Cr_15.4_Fe_13.7_Ni_17.7_Ta_22.1_Al_2.3_, at%), and the FCC matrix contains more Ni, Cr, Fe, and Al (Co_26.2_Cr_21.9_Fe_19.9_Ni_23.4_Ta_4.4_Al_4.2_, at%), Figure 4d.

The microstructural heterogeneity, i.e., the formation of non‐eutectic microstructures near the melt pool boundary, is attributed to the difference in kinetic mobility based on the current elemental constituents. Kinetic calculations using the commercial mobility database (Figure S9) indicate that the intrinsic diffusion coefficient of Ta (5.86 × 10^−9^ m^2^ s^−1^) in the Hyper‐HEA is around twice the values for Al (3.15 × 10^−9^ m^2^ s^−1^), Cr (2.03 × 10^−9^ m^2^ s^−1^), Fe (2.75 × 10^−9^ m^2^ s^−1^) Co (3.86 × 10^−9^ m^2^ s^−1^), and Ni (2.20 × 10^−9^ m^2^ s^−1^) at a temperature slightly above the melting point (1473 K). The difference in their kinetic mobility produces different extents of the solute trapping phenomenon during solidification.^[^ ^54^ ^]^ Figure 4e quantifies the solute trapping effect of Hyper‐HEA at different solid/liquid (S/L) interface velocities under the assumptions of the classic Aziz model.^[^ ^55^ ^]^ When the S/L velocity is close to 0, Ta, which has the highest diffusion coefficient among all existing alloying elements, diffuses from the S/L interface into the liquid. The concentration of Ta in liquid, on the other hand, stabilizes only when the S/L interface velocity reaches 0.3 m s^−1^. The concentration variations of the other elements due to solute trapping are less pronounced, which is consistent with the experimental observations (Figure S8). The rapid solidification at different regions in the melt pool is discussed individually because the S/L interface velocity is highly location‐dependent.^[^ ^56^ ^]^ The liquid near the bottom of the melt pool experiences Ta enrichment due to its extremely low S/L interface velocity. This is because Ta diffuses into the melt and yields Ta‐enriched spherical phases, as indicated in both the E‐HEA and Hyper‐HEA materials (Figure S5). While the trailing point on the top surface holds the same value as the laser scan speed,^[^ ^57^ ^]^ it is thus expected that with additional Al inputs (further into the hypereutectic regime), the remaining dendritic microstructures in Hyper‐HEA will be replaced by eutectic microstructures. It should be mentioned that it is challenging to eliminate the spherical Laves precipitates along the melt pool borders due to the rationales mentioned above.

For the mechanical properties, the microstructure observations (Figure 2) and hardness measurements (Figure 3) suggest that the eutectic lamellar structure is essential in enhancing the hardness and deformation resistance of the materials. These observations can be attributed to the additional hardening effect of the homogeneously distributed, nanosized Laves phase. The hardness values increase concurrently with the fraction of Laves phase in the current material class (Table S3). A similar trend is also observed in the EHEAs fabricated by conventional casting.^[^ ^58^ ^]^ The hardening contribution (Δσ_*y*_) of the Laves phase can be estimated according to: Δσ_*y*_ =  *k* · Δ*V_Laves_*,^[^ ^59^ ^]^ in which Δ*V_Laves_* is the fraction of the Laves phase, and *k* is 30–35 MPa at room temperature. The yield strength can be converted into hardness using the empirical Taylor relation.^[^ ^39^ ^]^ Based on this approximation, the additional hardening contribution from the extra fraction of Laves phase in Hyper‐HEA is ≈108 and 171 Hv compared to E‐HEA and Hypo‐HEA. These results mostly agree with the experimental hardness differences, i.e., 113 and 188 Hv. One possible explanation for the slight variation is the solid solution hardening of Al in the FCC phase. For instance, the Al concentration in the FCC phase in the Hypo‐HEA is 2.3 wt.% (Table S2), while the value in the Hyper‐HEA is ≈4.0–4.2 wt.% (Figure 4d).

**Figure 5:**
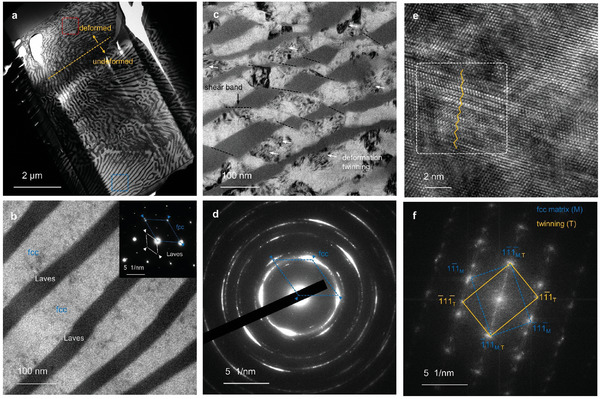
Deformation microstructure of the eutectic lamellar in the Hyper‐HEA. a) BF‐TEM micrograph showing the entire TEM specimen containing the deformed (*ε *= 50% in compression) and the undeformed regions. b) A higher magnification image from the undeformed region identical to the blue frame in (a). The inset shows an obtained SAED pattern from the TEM probing. c) Enlarged view of the red frame region in (a), showing the morphology of a severely deformed pillar (red dashed lines, shearing of the Laves phase; yellow arrows, defects in the deformed FCC matrix). d) Corresponding SAED pattern showing the refined microstructure after deformation, as indicated by the diffraction ring. e) HR‐TEM analysis showing twinning from the deformed FCC matrix. f) the Corresponding FFT pattern acquired from the white frame in (e) confirms the deformation twinning.

The topologically close‐packed Laves phase is typically considered extremely brittle at room temperature due to its high Peierls stress and a limited number of kinematically independent slip systems for dislocation motion.^[^ ^60^ ^]^ However, the current nanostructured Hyper‐HEA shows excellent micropillar compressive ductility (*ε *> 50%). We performed TEM analysis on the deformed sample to understand the underlying mechanism (**Figure** **5**a). The bright‐field (BF) transmission electron microscopy (TEM) image shows the absence of defects in the undeformed eutectic lamellae (Figure 5b). In contrast, the deformed pillar (Figure 5c) shows serrated Laves phases and heterogeneously spaced shear bands (red dashes) and mechanical twins (yellow arrows) within the FCC matrix. More specifically, the nanosized Laves phase was sheared and reoriented into small debris particles, with no cracks observed. This is different from the scenario for the micrometer‐sized Laves phase under plastic deformation, in which cracks are initiated within the Laves phase and then lead to the failure of the bulk materials.^[^ ^61^ ^]^ In addition, the deformed microstructure is significantly refined according to the selected area electron diffraction (SAED) pattern (Figure 5d). The high‐resolution (HR)‐TEM (Figure 5e) and fast Fourier transformation (FFT) pattern (see inset in Figure 5f) show that the defects observed in Figure 5c are deformation twins. This is also indicated by the serrations that appear in the flow stress curve (Figure 3f). Therefore, the remarkable micropillar compressive ductility is attributed to the 1) uniform distribution of the nano‐scaled lamellar colonies, which helps to avoid Laves phase cracking and reduces stress concentration points, hence mitigating damage initiation; and 2) the mechanical twinning activated at high strains in the FCC matrix phase, which equips the material with strain hardening during plastic deformation. The continuous work hardening behavior (Figure 3f) is thus attributed to the pinning of dislocations at phase boundaries, dynamic microstructure refinement due to precipitate shearing and twinning, and the higher dislocation storage capacity from deformation twinning. Overall, the ultrahigh hardness of the current as‐built Hyper‐HEA makes it a promising candidate to serve in precision instrument, molding, and drilling applications. Two strategies can potentially minimize and eliminate the non‐eutectic microstructures for further investigations. First, the fraction of melt pool boundaries and its associated spherical precipitates can be decreased by having larger melt pools. This can be achieved by switching from the LPBF production method to directed energy deposition with higher energy input. Second, replacing Ta with other elements with lower partitioning tendencies, e.g., W, Nb, Mo, can prevent the occurrence of segregation near the melt pool boundaries with the aid of the new EHEAs design toolbox.

## 4 Conclusion

We demonstrate here that the thermodynamically guided high‐throughput filtration alloy design strategy allows for the realization of EHEAs, with ultrahigh hardness and compatibility for rapid solidification fabrications. This is achieved by configuring and screening a preliminary dataset comprising 699840 HEAs spanning a wide compositional space. The new strategy is guided by the empirical formula derived from established experimental observations, thermodynamics computations, and the consideration of phase growth kinetics under rapid solidification (skewed phase diagram). Despite the minor cracks in the as‐built condition, the current Hyper‐HEA material contains a high fraction of eutectic lamellar nanostructure. It possesses an ultrahigh hardness of 675 ± 47 Hv with potential applications as hard coatings. In addition to this specific composition, the current alloy design toolbox accelerates the design of new EHEAs, especially under rapid solidification.

## 5 Experimental Section

### Composition Screening

The composition screening task was carried out using self‐written Python code. The (A_x1_A_x2_)(B_x3_B_x4_B_x5_B_x6_) formula mimics the B_2_A‐type Laves,^[^ ^62^ ^]^ and each element in the composition can take a stoichiometric coefficient of 0.1, 0.3, 0.5, 0.7, 0.9, or 1.0. By specifically selecting group B elements (Cr, Mn, Fe, Co, Ni) with similar mixing enthalpies and group A elements (Al, Zr, Ta) that possess highly negative mixing enthalpies with group B elements, there were in total 5*C*4*3*C*2* 6^6^ =  699840 possible alloy candidates. The atomic size mismatch (δ*r*) and average valence electron concentration (*VEC*) were computed according to $\delta r\;=\;100\%\sqrt{\sum c_{i}{\left(1-r_{i}/\bar{r}\right)}^{2}}$ and *VEC*  =  ∑*c_i_VEC_i_*.^[^ ^63^ ^]^ In which the *c_i_*, *r_i_*, *VEC_i_* were the atomic fraction, atomic radius, and valence electron concentration of element *i*, and $\bar{r}=\;\sum c_{i}r_{i}$ was the average atomic radius. These elemental intrinsic properties were taken from prior literature.^[^ ^64^, ^65^, ^66^ ^]^

Previous investigations show that the lower threshold of δ*r* for forming the Laves phase in high‐entropy alloys was ≈5.0%.^[^ ^32^ ^]^ As the δ*r* value increases, the likelihood of the appearance of an amorphous phase increases.^[^ ^67^ ^]^ Due to the estimative nature of such an empirical formula, the boundary of 4.9 < δ*r* < 5.1 was decidedto examine potential candidates in the vicinity of this lower threshold to prevent the occurrence of undesired amorphous phases. As for the valence electron concentration (VEC) indicator, it was inferred from previous computational data^[^ ^33^ ^]^ that Laves phase primary occurs in the range of 5.7 < VEC < 8.0 for equiatomic HEAs, and 5.6 < VEC < 9.3 for non‐equiatomic HEAs. Experiment results^[^ ^68^ ^]^ suggest that a lower VEC could yield a fully C14 alloy system. So far, the highest VEC value for the experimentally validated dual‐phase FCC‐Laves alloy was ≈8.0. An upper threshold of 8.0 confirmed by both computational and experimental results was selected and fixed a range of 7.9 < VEC < 8.1 for the VEC criteria.

The phase constituents of the 294 down‐selected compositions based on the empirical constraints were interpreted by ThermoCalc 2023b (TCHEA6, MobHEA3 databases),^[^ ^69^ ^]^ under the equilibrium condition, at their respective solidus temperatures. The elemental diffusion coefficients were assessed at 1473 K, slightly above the alloy's melting temperature. Solute trapping calculations were sequentially performed at each fixed S/L interface velocity (from 0 to 0.3 m s^−1^), and the elemental concentration within the liquid was recorded accordingly.

### Materials Processing

The pre‐alloyed hypoeutectic HEA powder was fabricated by gas atomization with high‐purity metals as ingredients (>99.9 wt.%) under an argon atmosphere. The chemical composition of the pre‐alloyed powder was analyzed using the wet‐chemical method, which was Co_26.6_Fe_18.7_Ni_23.3_Cr_19.8_Ta_9.3_Al_2.3_ (wt.%). Powders with a particle size distribution ranging from 25–60 µm were selected for the following LPBF fabrications. Additionally, pure Al powder (>99.9 wt.%) with a mean powder size of 20.8 µm was purchased from TLS Technik (Germany). The pre‐mixed powders were then prepared by blending the pre‐alloyed powder with different Al powder contents through a tumbler mixing machine (WAB AG, Switzerland). The powder mixtures were blended for 1 h at 30 rpm. The mixing ratio and nominal chemical composition of the powder mixtures were shown in Table S1. Before the experiments, all feedstock powders were dried at 373 K for 1 h to reduce moister contamination. All specimens were fabricated in a commercially available LPBF machine (Aconity MINI, Aconity3D, Germany). The machine had a laser spot size of 90 µm and a laser wavelength of 1064 nm. To assess the printability of the material, a wide range of processing conditions were tested on the hypo‐HEA pre‐alloyed powders. The processing parameters were laser power of 300 W, scanning speed ranging from 250 to 1050 mm s^−1^, hatch spacing of 100 µm, and layer thickness of 50 µm. By examining the as‐built sample's dimensional accuracy and the occurrence of large catastrophic cracks, the scanning speed of 750 mm s^−1^ was determined as the optimized processing condition. Given the similar chemical compositions among these three alloys, the same processing condition was thus adopted for E‐HEA and Hyper‐HEA. All productions were performed under an Ar atmosphere with an oxygen concentration <200 ppm. Specimens with a dimension of 10 × 10 × 4 mm^3^ were obtained for each composition with different parameter sets. Carbon steel substrate was adopted for all sample fabrications.

### Microstructural Characterization

The microstructure of the as‐built alloys was characterized using multiscale probing techniques. The crack propensity was analyzed using an optical microscope (OM, Zeiss Axioskop 2 MAT). Backscattered electron (BSE) imaging and electron backscatter diffraction (EBSD) measurements were conducted in a scanning electron microscope (SEM, Zeiss Sigma) operated at 30 kV. The EBSD data was analyzed using the OIM software (v8). Energy–dispersive X‐ray spectroscopy (EDS) mappings were carried out in the same SEM instrument with a dwell time of 100 µs for 256 frames operated at 15 kV. Backscattered electron (BSE) imaging analyses were performed by an SEM (Zeiss Merlin) operated at 30 kV. Atom probe tomography (APT, Cameca LEAP 5000 XR) was characterized in laser mode (pulse rate 125 kHz, pulse energy 40 PJ, temperature 60 K). Transmission electron microscopy (TEM) experiments, including bright‐field (BF), dark‐field (DF), and selected area electron diffraction (SAED), were carried out at 300 kV using a transmission electron microscope (JEOL JEM 2200).

### Mechanical Testing

The room temperature hardness values for all the specimens were measured on the polished surfaces using two types of hardness testers at different length scales. The first type of tester (Leco LM100AT) with a Vickers pyramidal diamond tip was used to probe the hardness response at the microscale, which was more representative of the overall hardness of the alloys. The maximum load was up to 500 g with a holding time of 13 s. The distance between adjacent indents is 300 µm, and the hardness value was averaged using at least ten indents. Another type of tester (G200, Keysight‐Tec, USA) with a diamond nanoindenter (pyramidal shape) was used to measure the hardness value within individual grains. The nanoindents with a spacing of 10 µm were placed based on the average grain size to cover more grains and avoid the overlap of the indentation‐induced deformation zones. The measurement was performed under a constant loading rate (0.05 s^−1^) up to a maximum displacement of 500 nm. A micro‐compression study (Hysitron TI950 nanoindenter) was conducted with micro‐pillar samples at a strain rate of ≈1 × 10^−3^ s^−1^. The pillars were prepared with the dual FIB‐SEM system (FEI Helios Nano‐Lab 600i) under identical conditions with a diameter of 3 µm, a height of 6 µm and a taper angle < 2° (The height/diameter ratio is kept ≈2).

## Conflict of Interest

The authors declare no conflict of interest.

## Author Contributions

L.H., Z.S., and Y.L. designed the research project; Z.S., P. W., and A.C.Y.N. performed the high‐throughput alloy composition screening and Calphad calculations. L.H. and X.Z. characterized the alloys; W.X. and J.R. performed the micropillar compression and nanoindentation experiments. L.H., Z.S., and S.T. analyzed the data; L.H., Z.S., Z.L., and D.R. conceptualized the paper; all authors contributed to the discussion of the results.

## Supporting information

Supporting Information

## Data Availability

The data that support the findings of this study are available from the corresponding author upon reasonable request.
