# Supplementary material for: Thermodynamics‐Guided High‐Throughput Discovery of Eutectic High‐Entropy Alloys for Rapid Solidification
Source: Adv Sci (Weinh). 2024 Jun 18;11(31):2401559. doi: 10.1002/advs.202401559 (PMC11336915; doi:10.1002/advs.202401559)
Supplement: Supplementary file 1 — Supporting Information [file ADVS-11-2401559-s001.docx]

Supplementary information for

**Thermodynamics-guided high-throughput discovery of eutectic high-entropy alloys for rapid solidification**

Liuliu Han^1^, Zhongji Sun^2*^, Wenzhen Xia^3^, Shao-Pu Tsai^4^, Pei Wang^2^, Andrew Chun Yong Ngo^2^, Yong Liu^5*^, Zhiming Li^6^, Dierk Raabe^1^

*^1^Max-Planck-Institut für Eisenforschung, Max-Planck-Straße 1, 40237 Düsseldorf, Germany*

*^2^Institute of Materials Research and Engineering, Agency for Science, Technology and Research, 138634, Singapore*

*^3^School of Metallurgical Engineering, Anhui University of Technology, Maanshan 243002, China*

*^4^Department of Materials Science and Engineering, National Taiwan University, 10617 Taipei, Taiwan*

*^5^State Key Laboratory of Powder Metallurgy, Central South University, 410083 Changsha, China*

*^6^School of Materials Science and Engineering, Central South University, 410083 Changsha, China*

**Correspondence to: sun_zhongji@imre.a-star.edu.sg; yonliu@csu.edu.cn*

**Supplementary text**

Fig. S1a and S1b display the cross-sectional microstructure of the pre-alloyed hypoeutectic powder in electron backscatter diffraction (EBSD) and backscattered electron (BSE) imaging mode. The powder comprises a typical eutectic lamellar structure with small hypo- (red arrows) and hypereutectic (blue arrows) regions. By averaging over multiple images, the volume fractions of these three regions are estimated to be 95%, 3% and 2%, respectively. Microstructural heterogeneity is mainly due to the non-equilibrium nature of rapid solidification during gas atomization. As the outer regions of the atomized powders experience a different set of cooling conditions in comparison to their inner cores, different microstructures will be yielded. Moreover, inhomogeneous mixing among the elements within liquid will also produce highly heterogeneous microstructures. Fig. S1c shows the electron backscatter diffraction (EBSD) analysis of the typical eutectic microstructure within the orange frame in Fig. S1b. The BSE image (Fig. S1b) and phase map (Fig. S1c top) show that the bright lamellar rods in the BSE image are the Laves phase while the remaining darker areas are the FCC matrix phase. The corresponding inverse pole figure (IPF) maps (Fig. S1a and S1c bottom) reveal that the powder has an average eutectic cell (where the FCC phase has the same orientation) size of 4.3 ± 2.8 μm. The atomic-scale chemical compositions of the Laves and FCC phases are examined by atom probe tomography (APT, Fig. S1d). The interface between the FCC and Laves phases is highlighted in terms of a 10 at.% Ta isocomposition surface. Fig. S1e shows the one-dimensional (1D) compositional profiles obtained along the red arrow in Fig. S1d. Co and Ta enrichment was observed at the FCC/Laves interface, accompanied by a slight depletion of Fe, Cr and Ni (black dashed frame in Fig. S1e). The chemical compositions of the FCC and Laves phases averaged from 3 APT datasets are determined as Co_27.0_Ni_25.3_Cr_20.3_Fe_18.9_Ta_6.2_Al_2.3_(at.%) and Co_27.2_Ni_19.8_Cr_17.8_Fe_15.1_Ta_18.6_Al_1.5_ (at.%), respectively. To systematically understand the eutectic variants, two additional alloy compositions were made by mixing the hypoeutectic pre-alloyed powder with additional pure aluminium powders (see illustrations in Fig. S2).


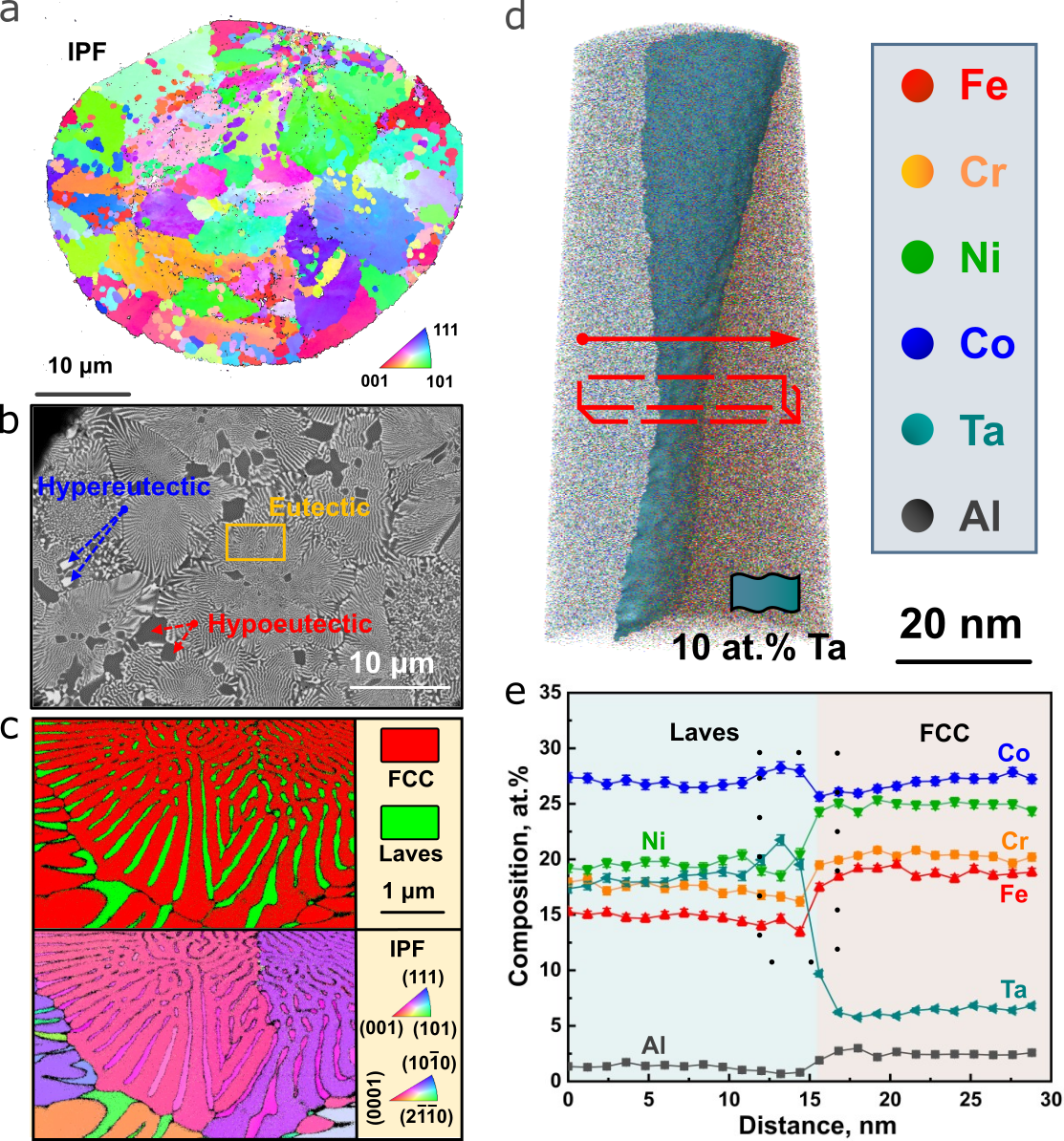


**Figure S1.** **a,** Cross-sectional IPF map of the pre-alloyed hypoeutectic Co_26.6_Fe_18.7_Ni_23.3_Cr_19.8_Ta_9.3_Al_2.3_ (at.%) powder. **b,** Cross-sectional BSE image of the pre-alloyed hypoeutectic powder. **c,** Corresponding EBSD phase and IPF maps (orange rectangle in **b**). **d,** APT map exhibiting the elemental distributions of the FCC and Laves phases in the eutectic region. **e,** 1D compositional profiles across the phase boundary (along the red arrow in **d**). The black dash frame illustrates the elemental segregation near the FCC/Laves interface.


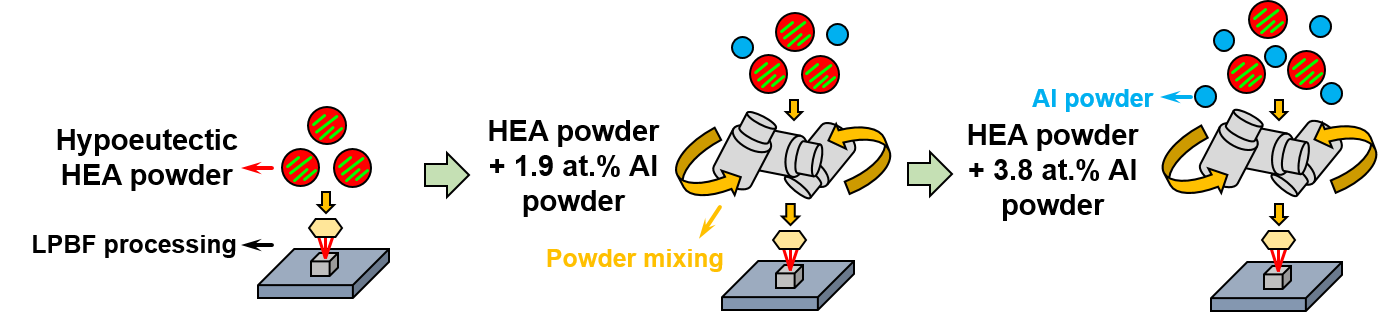


**Figure S2.** **Schematic illustration of the powder preparation procedures**


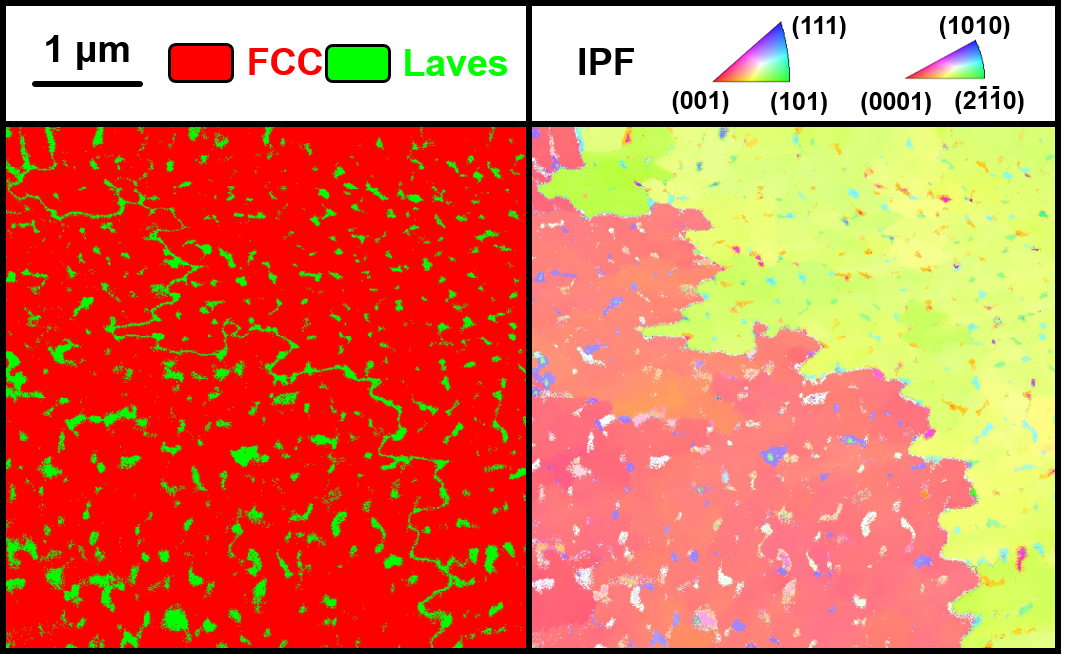


**Figure S3. High-resolution EBSD analysis showing the dendritic and interdendritic structures in the Hypo-HEA.** According to the phase and IPF maps, the dendritic and interdendritic structures are FCC and Laves phases, respectively.


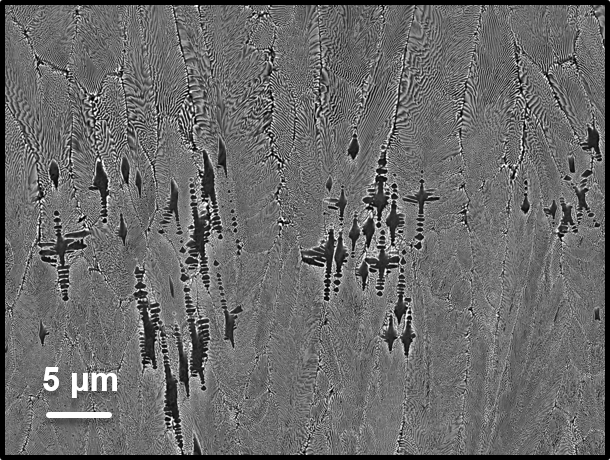


**Figure S4. Electron channeling contrast imaging (ECCI) showing the remaining dendritic regions in the Hyper-HEA.**


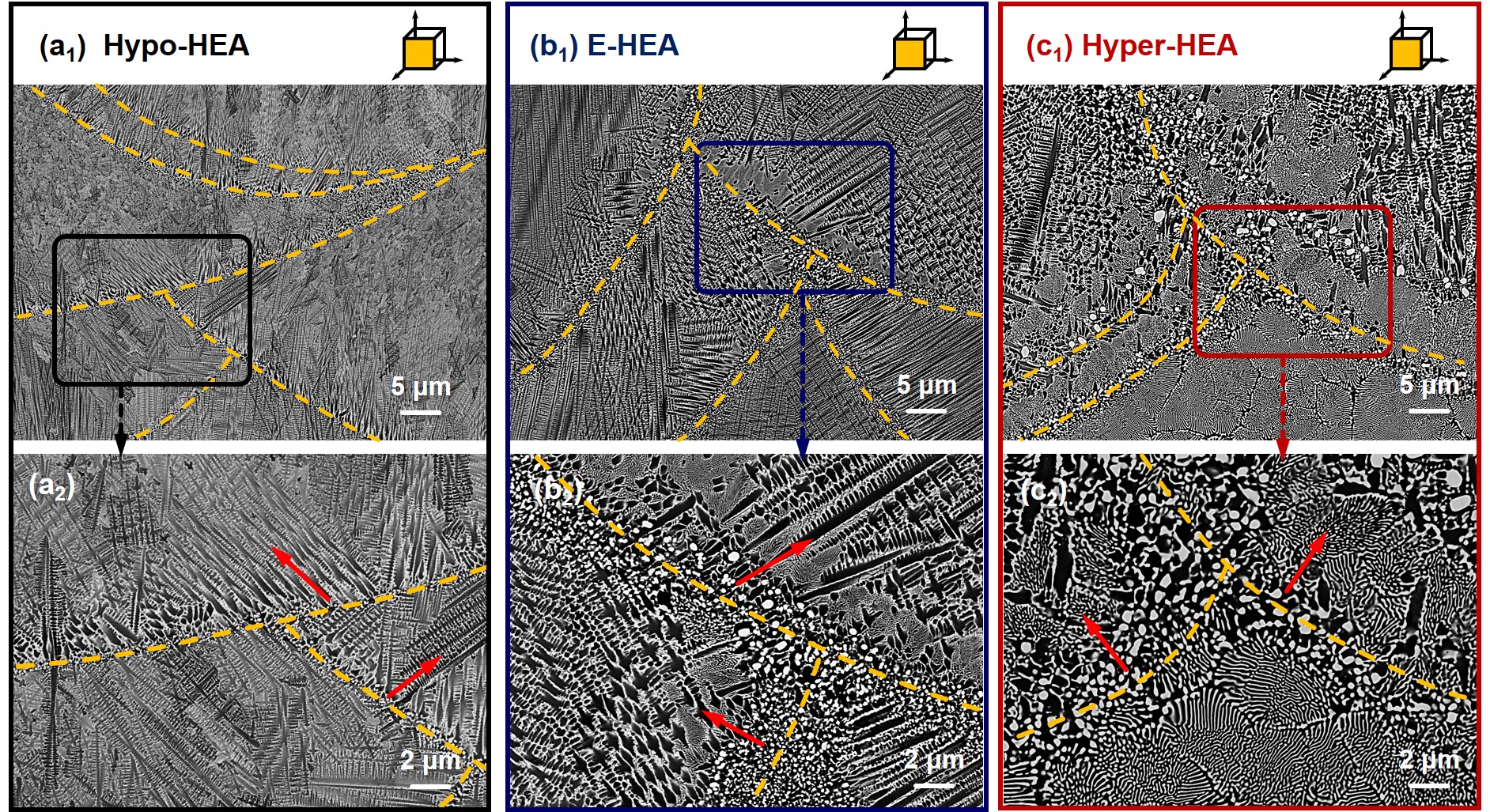


**Figure S5. BSE images showing the side view** **microstructures of the as-built HEAs.** (**a_1_, a_2_**) Hypo-HEA, (**b_1_, b_2_**) E-HEA and (**c_1_, c_2_**) Hyper-HEA. (**a_2_, b_2_, c_2_**) are the enlarged views taken from the framed regions in (**a_1_, b_1_, c_1_**). The orange dashed lines mark the melt pool boundaries.


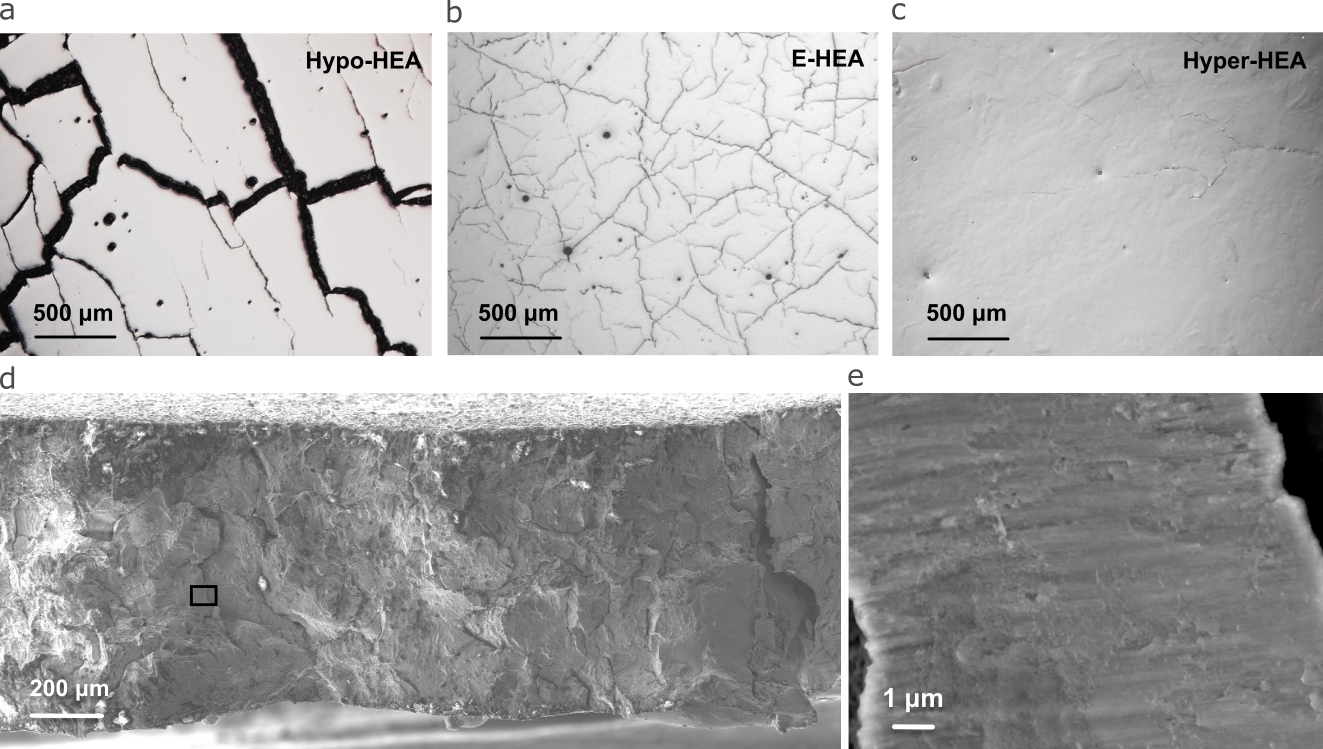


**Figure S6. a, b,** and **c,** Typical optical images of the LPBF-built alloys with different Al contents. **d,** the fracture surface of the Hypo-HEA with its enlarged view in **e**. The smooth crack surface indicates the presence of hot (aka solidification) crack.


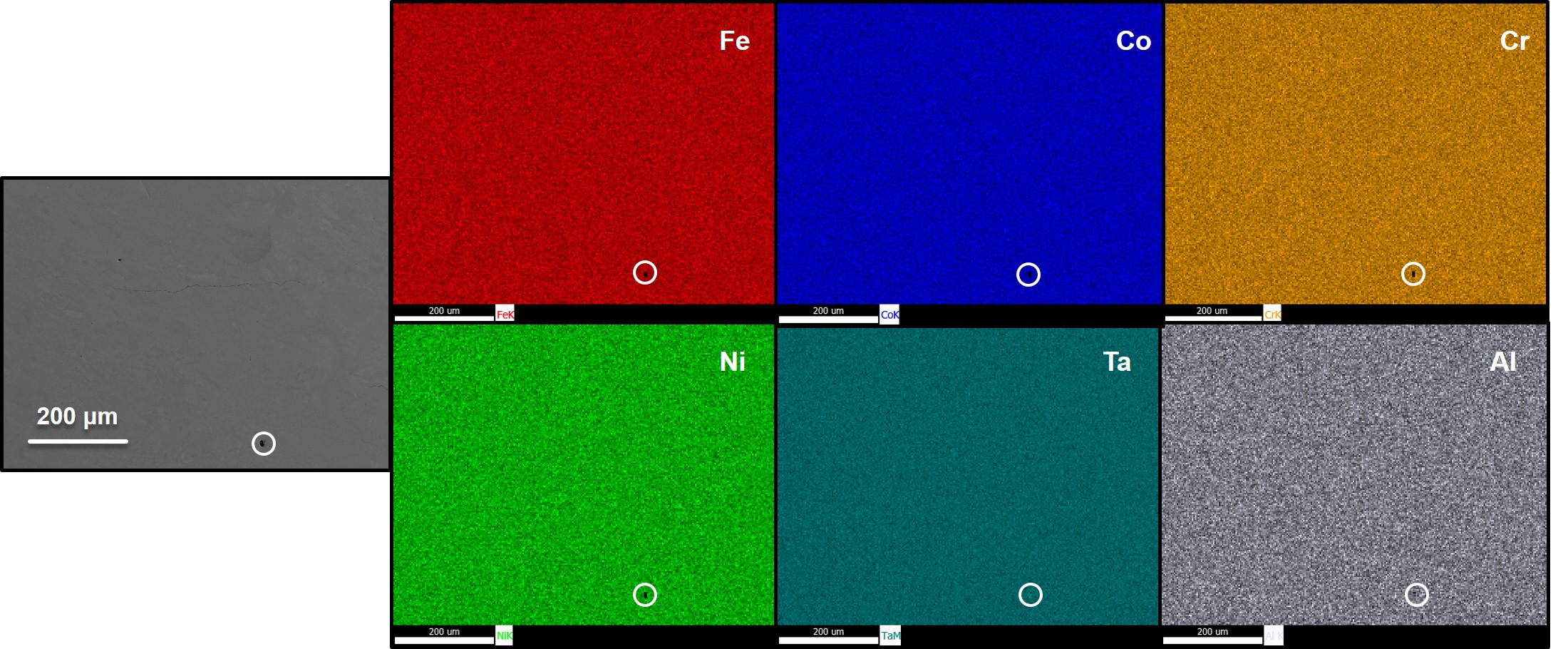


**Figure S7. SEM image and corresponding EDS elemental distribution maps of the Hyper-HEA.** The white circle marks a pore.


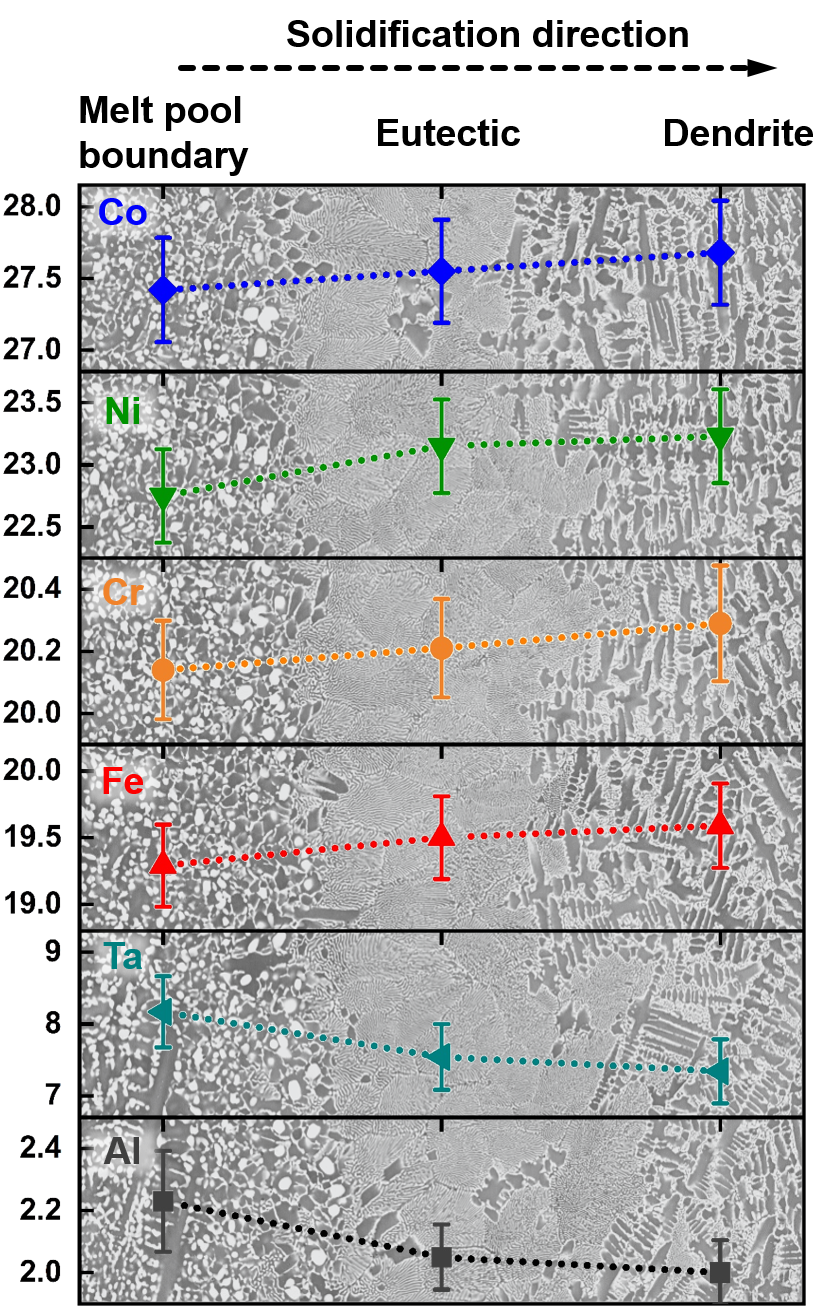


**Figure S8.** Plot of the average elemental concentrations derived from the EDS mappings in Fig. 4.


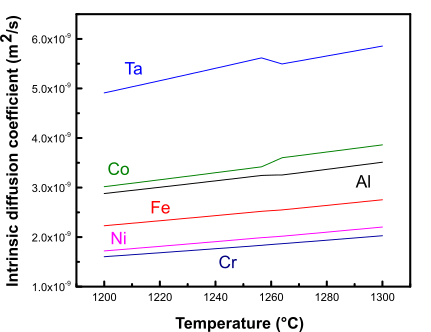


**Figure S9. Intrinsic diffusion coefficients of all alloying elements within the liquid phase of the Hyper-HEA between 1200 °C and 1300 °C.** Computed from ThermoCalc 2023b software with TCHEA6 and MobHEA3 databases.

**Table S1.** 18 alloys fulfill the requirements of (a) only consist of face-centred cubic (FCC) and Laves phases, (b) and have a Laves molar fraction above 0.2 from the initial 699840 compositions.

|  | VEC | δr | Composition | Solidus Temperature | Phase Number | Phase_01 | Phase_01_Amount | Phase_02 | Phase_02_Amount |
| --- | --- | --- | --- | --- | --- | --- | --- | --- | --- |
| 1 | 8.03 | 5.09 | Cr0.5Fe0.9Co0.7Ni0.9Al0.1Ta0.3 | 1260.28 | 2 | FCC_L12#1 | 0.79 | C14_LAVES | 0.21 |
| 2 | 8.03 | 5.07 | Cr0.7Fe0.5Co0.9Ni1.0Al0.1Ta0.3 | 1241.76 | 2 | FCC_L12#1 | 0.79 | C14_LAVES | 0.21 |
| 3 | 8.00 | 4.98 | Cr0.5Fe1.0Co1.0Ni0.7Al0.1Ta0.3 | 1276.65 | 2 | FCC_L12#1 | 0.80 | C14_LAVES | 0.20 |
| 4 | 8.00 | 5.04 | Cr0.5Fe0.9Co1.0Ni0.7Al0.1Ta0.3 | 1273.16 | 2 | FCC_L12#1 | 0.79 | C14_LAVES | 0.21 |
| 5 | 8.00 | 5.07 | Cr0.7Fe0.5Co1.0Ni0.9Al0.1Ta0.3 | 1247.94 | 2 | FCC_L12#1 | 0.78 | C14_LAVES | 0.22 |
| 6 | 8.00 | 4.94 | Cr0.7Fe0.7Co1.0Ni0.9Al0.1Ta0.3 | 1254.72 | 2 | FCC_L12#1 | 0.80 | C14_LAVES | 0.20 |
| 7 | 7.97 | 4.99 | Cr0.7Fe0.7Co0.9Ni0.9Al0.1Ta0.3 | 1252.86 | 2 | FCC_L12#1 | 0.79 | C14_LAVES | 0.21 |
| 8 | 7.97 | 5.02 | Cr0.5Fe1.0Co0.9Ni0.7Al0.1Ta0.3 | 1276.69 | 2 | FCC_L12#1 | 0.79 | C14_LAVES | 0.21 |
| 9 | 7.97 | 5.03 | Cr0.7Fe0.7Co0.7Ni1.0Al0.1Ta0.3 | 1242.73 | 2 | FCC_L12#1 | 0.79 | C14_LAVES | 0.21 |
| 10 | 7.97 | 5.09 | Cr0.5Fe0.9Co0.9Ni0.7Al0.1Ta0.3 | 1273.05 | 2 | FCC_L12#1 | 0.78 | C14_LAVES | 0.22 |
| 11 | 7.94 | 5.01 | Cr0.9Fe0.3Co1.0Ni1.0Al0.1Ta0.3 | 1235.73 | 2 | FCC_L12#1 | 0.78 | C14_LAVES | 0.22 |
| 12 | 7.92 | 4.93 | Cr0.9Fe0.5Co0.9Ni1.0Al0.1Ta0.3 | 1238.8 | 2 | FCC_L12#1 | 0.79 | C14_LAVES | 0.21 |
| 13 | 7.92 | 4.95 | Cr0.7Fe0.9Co0.7Ni0.9Al0.1Ta0.3 | 1255.04 | 2 | FCC_L12#1 | 0.79 | C14_LAVES | 0.21 |
| 14 | 7.92 | 4.93 | Cr0.7Fe1.0Co0.5Ni1.0Al0.1Ta0.3 | 1247.12 | 2 | FCC_L12#1 | 0.80 | C14_LAVES | 0.20 |
| 15 | 7.91 | 5.00 | Cr0.7Fe0.9Co0.5Ni1.0Al0.1Ta0.3 | 1242.55 | 2 | FCC_L12#1 | 0.79 | C14_LAVES | 0.21 |
| 16 | 7.91 | 5.06 | Cr0.9Fe0.3Co0.9Ni1.0Al0.1Ta0.3 | 1232.21 | 2 | FCC_L12#1 | 0.77 | C14_LAVES | 0.23 |
| 17 | 7.91 | 5.09 | Mn0.9Fe0.7Co1.0Ni0.5Al0.1Ta0.3 | 1133.67 | 2 | FCC_L12#2 | 0.80 | C14_LAVES | 0.20 |
| 18 | 7.91 | 5.08 | Cr0.7Fe0.7Co0.7Ni0.9Al0.1Ta0.3 | 1246.93 | 2 | FCC_L12#1 | 0.77 | C14_LAVES | 0.23 |

**Table S2.** The mixing ratio of the as-mixed alloys and the nominal chemical compositions.

|  | Pre-alloyed powder  (wt.%) | Al powder  (wt.%) | Nominal composition  (at.%) |
| --- | --- | --- | --- |
| Hypo-HEA | 100 | 0 | Co_26.6_Fe_18.7_Ni_23.3_Cr_19.8_Ta_9.3_Al_2.3_ |
| E-HEA | 99.25 | 0.75 | Co_26.1_Fe_18.3_Ni_22.9_Cr_19.4_Ta_9.1_Al_4.2_ |
| Hyper-HEA | 98.5 | 1.5 | Co_25.6_Fe_17.9_Ni_22.4_Cr_19.1_Ta_8.9_Al_6.1_ |

**Table S3.** The volume fraction of different microstructural features and phases in the as-built alloys. The results are based on the backscattered electron (BSE) and electron backscattered diffraction microscopy examinations (EBSD) analysis.

|  | Dendrite  (Vol.%) | Eutectic lamella  (Vol.%) | Anomalous precipitate  (Vol.%) | FCC  (Vol.%) | Laves  (Vol.%) |
| --- | --- | --- | --- | --- | --- |
| Hypo-HEA | 96.5 | 0 | 2.5 | 73.1 | 26.9 |
| E-HEA | 48.5 | 45.6 | 3.9 | 71.0 | 29.0 |
| Hyper-HEA | 10.2 | 85.5 | 4.3 | 68.2 | 31.8 |
